# Classroom, club or collective? Three types of community-based group intervention and why they matter for health

**DOI:** 10.1136/bmjgh-2020-003302

**Published:** 2020-12-15

**Authors:** Lu Gram, Sapna Desai, Audrey Prost

**Affiliations:** 1 Institute for Global Health, Department of Population Health Sciences, University College London, London, UK; 2 Population Council India, New Delhi, Delhi, India

**Keywords:** health policy, public health, child health, maternal health, health education and promotion

## Abstract

Interventions involving groups of laywomen, men and adolescents to promote health are increasingly popular, but past research has rarely distinguished between different types of intervention with groups. We introduce a simple typology that distinguishes three ideal types: *classrooms, clubs* and *collectives*. Classrooms treat groups as a platform for reaching a population with didactic behaviour change strategies. Clubs seek to build, strengthen and leverage relationships between group members to promote health. Collectives engage whole communities in assuming ownership over a health problem and taking action to address it. We argue that this distinction goes a long way towards explaining differences in achievable health outcomes using interventions with groups. First, classrooms and clubs are appropriate when policymakers primarily care about improving the health of group members, but collectives are better placed to achieve population-level impact. Second, classroom interventions implicitly assume bottleneck behaviours preventing a health outcome from being achieved can be reliably identified by experts, whereas collectives make use of local knowledge, skill and creativity to tackle complexity. Third, classroom interventions assume individual participants can address health issues largely on their own, while clubs and collectives are required to engender collective action in support of health. We invite public health researchers and policymakers to use our framework to align their own and communities’ ambitions with appropriate group-based interventions to test and implement for their context. We caution that our typology is meant to apply to groups of laypeople rather than professionalised groups such as whole civil society organisations.

**Summary box**

- Community groups of laypeople are a popular policy tool for health promotion.
- Past research has rarely distinguished between types of group-based interventions.
- We introduce a new typology: classroom, club or collective.
- Our typology helps to explain why not all health interventions with groups work.
- The typology provides a guide to choosing community group interventions in relation to context and outcomes.

## Introduction

It is a truth universally acknowledged, but all too often ignored, that not all community health interventions are the same. Since the Alma Ata Declaration, public health researchers, practitioners and policymakers have paid attention to the potential of interventions involving community groups to promote health in low- and middle-income settings.^1–3^ Unfortunately, researchers have often failed to distinguish between the manifold types of group-based intervention that exist,^4–6^ resulting in potential mismatch between intervention types and programme goals. We recently did a systematic review of the impact of women’s groups interventions on health in India which found a clear difference in health impact by intervention type, with interventions building community capacity more likely to achieve changes in population-level health outcomes than those simply offering information.^7^ Our present paper builds on this review to offer conceptual tools for thinking through observed differences in health impacts by type of group intervention. We introduce a typology of group interventions and argue that implicit assumptions underpinning different types of intervention matter for their likely health impact. Our typology is meant to apply to interventions involving groups of laypeople who meet on a regular basis rather than professionalised groups such as whole civil society organisations.

Interventions with groups of laywomen, men and adolescents have improved health outcomes across many health domains, including maternal and newborn health,^8^ injury and violence,^9^ non-communicable disease,^10^ sexually transmitted^11^ and other infectious diseases.^12^ For example, in Nepal, India, Malawi and Bangladesh, trained peer facilitators leading groups through a cycle of prioritising, planning and implementing strategies to address perinatal health problems reduced neonatal mortality by 20%.^8^ In South Africa, groups of women and men addressed the risk of HIV infection and intimate partner violence through microfinance, gender training and community mobilisation.^13^

Health interventions with groups have also enjoyed widespread policy support. India’s National Health Mission, National AIDS Control Program and National Rural Livelihoods Mission implement a range of women’s group interventions to improve maternal and child health, nutrition and HIV-related outcomes.^14–17^ Ethiopia’s Women’s Development Army has worked with groups to improve maternal health for over 15 years.^18^ Nigeria aims to tackle gender-based violence through livelihoods-based women’s affinity groups.^19^

The enthusiasm for group-based health interventions has not always been matched with a recognition of major differences across types of intervention,^4–6^ as implementers continue to refer to deceptively simple short-hands such as ‘health education’ or ‘community mobilisation’ with little detail about the specific techniques employed.^20^ Yet intervention type matters, because different, at times conflicting, assumptions about health and behaviour change underlie different types of group intervention.

For example, social and behaviour change experts recommend spending maximum time delivering key messages concerning a few well-defined behaviours.^21^ Targeting a small number of specific behaviours is thought to allow planners to tailor techniques and messages for maximum impact.^21^ Seeking to influence too many behaviours is seen as counterproductive. Learners may fail to understand, accept or remember all the information they are shown,^22^ and intervention designers may struggle to understand or address the drivers for a plethora of behaviours.^21^ Thus, this approach hopes instead to identify a limited set of bottleneck behaviours standing in the way of significant health gains.

Others argue the above approach is reductionist and misguided and may not be effective.^23^ Improving population-level health may be less akin to baking a cake, where following a recipe guarantees a good outcome, and more akin to raising a child, where every situation is unique, previous success is no guarantee of future success and expertise may not always help.^24^ Interventions seeking to improve health at national or even subnational scales may need to cover so many different social and epidemiological contexts that it becomes prohibitively expensive to design and test bespoke behaviour change techniques for every context.^25^ Instead, it may be more effective to spend group meetings engaging community members’ own knowledge of what health problems matter and how they can be addressed.^3^

While interventions can and do in practice combine multiple approaches to achieve health outcomes, implementers do not have unlimited access to time, material and human resources to achieve impact. Community members may be neither able nor motivated to attend endless meetings or contribute to community projects without obvious benefit.^26^ The poorest of the community who can least afford to forego time spent on wage or domestic labour are most at risk of being excluded.^27^ Thus, decision-makers must prioritise the most effective actions—whether they are delivery of key messages or bottom-up community action. We present a simple typology of community-based health interventions with groups to serve as a practical guide for researchers and policymakers.

## A typology of health interventions with groups

**Figure 1:**
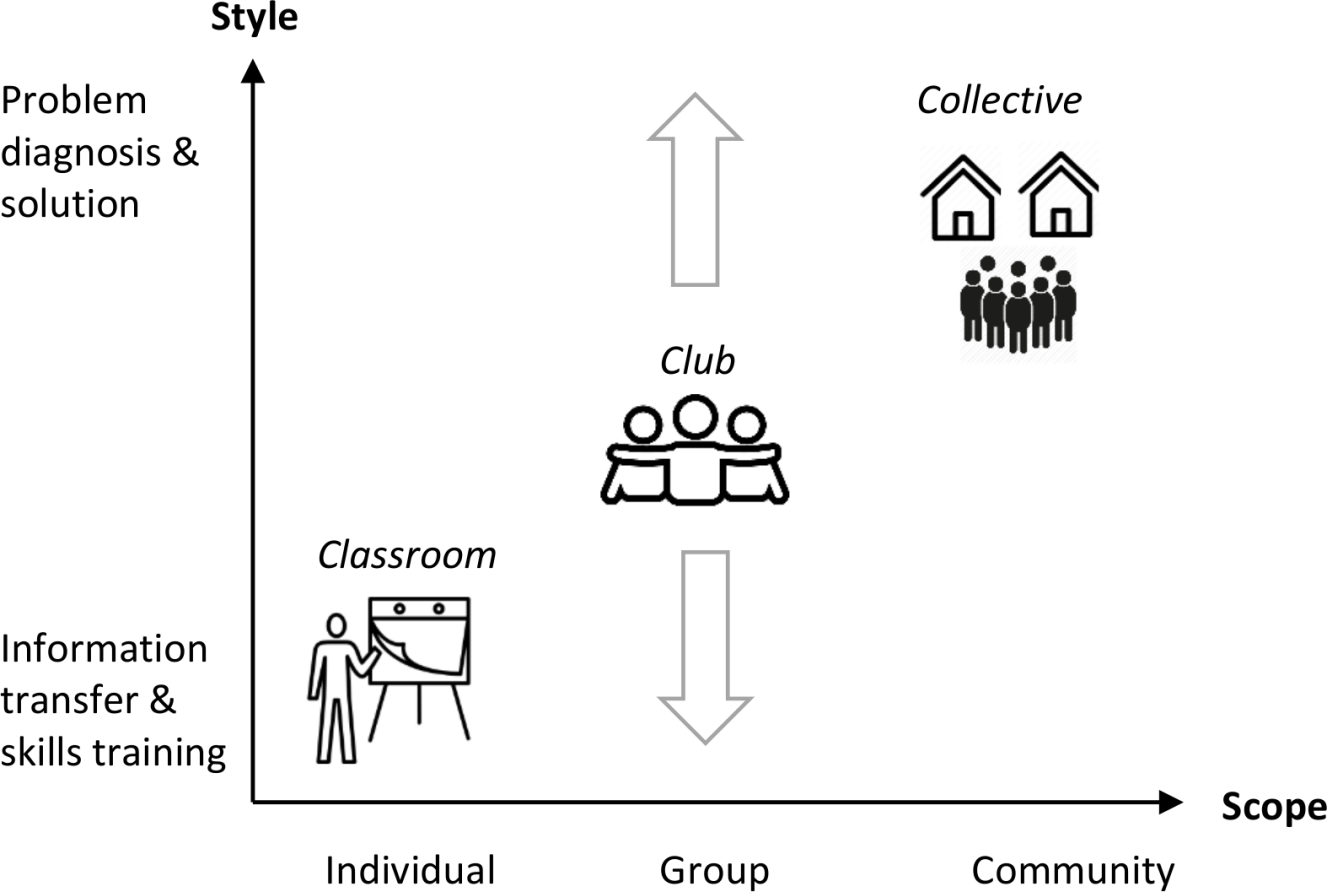
Characteristics of health interventions with groups.

We distinguish two critical axes of variation among interventions with groups (figure 1): style and scope of intervention activities. The axes represent continua rather than discrete categories. Interventions placed higher up on the *y*-axis devote a greater share of time, material and human resources to problem diagnosis and solution rather than information transfer and skills training. Interventions placed further along the *x*-axis focus on strengthening and leveraging community over individual capacity for health. Thus a continuum of interventions exists along either axis.

*Style* refers to intervention implementers’ approach to health communication. Didactic styles focus on content delivery, information transfer and skills training. Problem-solving styles engage group or community members in diagnosing the causes of health problems and encourage them to devise solutions. *Scope* refers to the size of the social unit targeted by the intervention for capacity strengthening. Interventions building individual capacity use groups primarily as a logistical convenience for reaching large numbers of individuals with health messages. Interventions having group capacity as their scope build, strengthen and leverage interpersonal relationships between group members for health purposes. Interventions with a community-wide scope build community capacity.^28 29^ They engage the whole community in assuming ownership over addressing a health problem, that is, developing a collective sense among community members that the health problem is ‘ours’ to address, rather than ‘somebody else’s problem’.^30^

Using our two axes of variation, we can now define three ideal types of health intervention with community-based groups: the classroom, the club and the collective. The *classroom* is a health intervention using didactic pedagogy to transfer knowledge and/or skills to a group of individuals with a focus on building individual capacity rather than group or community capacity. The *club* is an intervention that builds, strengthens and leverages the capacity of groups to improve health among members, but spends minimal effort in expanding or leveraging the capacity of the wider community to this end. We define club interventions purely in terms of their scope, so they may employ either didactic or problem-solving styles. *Collectives* are group-based health interventions that combine a community-wide scope with a problem-solving style.

A number of examples will help clarify these terms (table 1). A *classroom* intervention in Tanzania involved a trained teacher holding video screening for groups of school children followed by the distribution of leaflets to each pupil to raise awareness of risks of tapeworm infection.^31^ A didactic *club* intervention in India delivered maternal and child health messages to financial self-help groups through a peer educator, leveraging social cohesion among group members to encourage them to support each other in seeking maternal care from health providers.^32^ As an example of a problem-solving *club* intervention, village health clubs in Zimbabwe engaged group members in developing strategies to address local priorities,^33^ but interacted little with non-club members in this process. Women’s groups practising participatory learning and action are an example of a *collective*.^8^ Other examples include interventions to prevent HIV infection and intimate partner violence in South Africa^13^ and sex worker collectives promoting sexual health in urban India.^34^

**Table 1:** Example interventions for each intervention type

Intervention type	Style	Scope	Intervention design
Classroom	*Didactic*. Intervention primarily sought to transfer information about worm infection to its target audience.	*Individual*. Classrooms were targeted mainly as a logistical convenience for reaching large numbers of children.	Video screening for school children about tapeworm infection in Tanzania followed by distribution of information leaflets.^31^
Club (didactic)	*Didactic*. Focus on delivering maternal and child health messages. Discussion was only used to check for women’s understanding of these health messages.	*Group*. Social cohesion among group members facilitated the emergence of mutual social support, for example, in seeking maternal care from health providers.	Health education for members of financial self-help groups regarding maternal and newborn care and care-seeking practices in India.^32^
Club (problem-solving)	*Problem-solving*. Club members developed strategies to address local priorities, ending each session with a pledge to do homework before the next weekly session.	*Group*. Club members visited each other to provide support and monitor progress on homework. However, they interacted little with outside non-club members in this process.	Village Health Clubs stimulating behaviour change and demand for improved water, hygiene and sanitation in Zimbabwe.^33^
Collective	*Problem-solving*. Group facilitators lead members through a cycle of prioritising, planning and implementing their own strategies to address health problems.	*Community*. Group members involve the wider community in staffing bicycle ambulances, producing and distributing clean delivery kits or lobbying local government.^71^	Women’s groups practising participatory learning and action to promote maternal and newborn health in Malawi, Nepal, India and Bangladesh.^8^

It is worth noting that many interventions labelled ‘interactive’ or ‘participatory’ in the health literature are still ‘didactic’, even if they engage group members in naming fruits on a flip chart or singing slogans about healthy behaviours.^35^ Such activities move beyond pure knowledge transfer and seek to motivate participants to enact target health behaviours, but do not meaningfully involve participants in choosing priorities or methods for health improvement. ‘Problem-solving’ styles also do not preclude group facilitators introducing participants to a variety of topics and heath behaviours, only little effort is involved in designing and delivering these messages compared with that of engaging participants in problem diagnosis and strategy formulation.

Finally, as is evident from figure 1, other forms of intervention apart from classrooms, clubs and collectives are theoretically possible. For example, a pilot intervention combined an individual scope with a problem-solving style by offering groups of female sex workers training in cognitive reframing to enhance their sense of individual self-worth and agency.^36^ Another intervention combined a community-wide scope with a didactic style by using street theatre, video screenings, poster campaigns, neighbourhood pledges and school visits to change social norms around hygiene and sanitation.^37^ To our knowledge, such examples are rare among interventions with community-based groups.

## Intervention types and health outcomes

Intervention type matters, because not all types suit all contexts and outcomes. Four critical questions should be considered before choosing a type of intervention:

1. Does the intervention only aim to improve group members’ health or is it intended to achieve population-level impact?
2. Does achieving the health outcome require social support or collective action from group or community members?
3. Can intervention designers reliably identify a limited set of bottleneck behaviours preventing the health outcome from being achieved?
4. Do group or community members have sufficient time and willingness to reflect, innovate and adapt to solve their health problems together with a facilitator?

Table 2 shows how answers to these questions might reveal preferred intervention types. In the last column, we provide examples from the literature on public health interventions with groups to illustrate outcomes that were successfully modified through each type.

**Table 2:** Mapping group interventions to contexts and health outcomes: characteristics and assumptions

Intervention type	Relevant target population	Assumption 1: *Support*	Assumption 2: *Behaviours*	Assumption 3: *Problem-solving*	Examples of outcomes achieved
Classroom	Individual group members	Individuals can change risk factors largely on their own	Bottleneck behaviours and their enablers and barriers can reliably be identified by experts	Few problem-solving capacities are required; group members only need to follow instructions	Improved knowledge of and attitudes towards tapeworm infection among targeted school children^31^
Club (didactic)	Group members	Support from staff and group members suffice to change risk factors	As above	As above	Improved uptake of antenatal and postnatal care services among group members^32^
Club (problem-solving)	Group members	As above	Bottleneck behaviours are disputed or unknown to experts	Group members are willing and able to collectively reflect, innovate and adapt to address health issues with facilitators	Greater utilisation of latrines among club members^33^
Collective	The general population	Collective action by staff, group members and the wider community is needed to change risk factors	As above	Group and community members are willing and able to reflect, innovate and adapt to address health issues with facilitators	Improved population-level rates of newborn survival^8^

*Does the intervention only aim to improve group members’ health or is it intended to achieve population-level impact?* Classroom and club interventions have historically been aimed at improving the health of group members rather than population-level outcomes. Evaluations of such interventions have exclusively assessed impact on group members rather than the general community.^33 35 38^ Evaluations of collectives such as women’s groups practising participatory learning and action to prevent neonatal mortality, microfinance groups combined with community mobilisation to prevent intimate partner violence or sex worker collectives to prevent HIV/STI (sexually transmitted infection) have all assessed population-level impacts.^8 13 39^

Collectives involve the whole community in achieving population-level impact through mechanisms such as agitating for social norm change, altering the natural and built environment or influencing local governance.^3^ However, classrooms and clubs have no intentional mechanisms for involving the wider community. It is in theory possible that group members might improve population-level health outcomes by spontaneously diffusing knowledge and skills to the community. In practice, there is little evidence of this happening.^40^ Community health researchers emphasise the importance of ‘organised diffusion’, that is, active involvement of intervention staff in encouraging dissemination of knowledge and skills to the wider community, to ensure it happens.^41 42^

It is also theoretically possible that so many community members join groups that everyone becomes a group member. We do not see this often in practice either. Across seven trials of women’s groups practising participatory learning and action in Bangladesh, India, Nepal and Malawi, implementers made extensive efforts to maximise group attendance by going door-to-door to invite and persuade women to attend each meeting, flexibly adjusting meeting times to suit the wishes of women themselves and deliberately holding meetings close to hard-to-reach community members.^43^ Even so, no intervention ever managed to get more than half of the target population to attend one or more meetings, while median attendance was just 37%.^8^

*Does achieving the health outcome require social support or collective action from group or community members?* Classroom interventions assume that risk factors influencing the health outcome can be modified by participants themselves without much assistance from other group or community members. This works for easily recognisable, immediate threats that require individuals to alter a single conscious, planned behaviour,^44^ but many types of behaviour change require external support, such as behaviours that call for individuals to break habits (for example, giving up smoking), respond to emergencies (delivering in a hospital) or expend time and financial resources (visiting an antenatal clinic).^45^ New technologies, restrictive policies or cash incentives may facilitate such behaviours, but are not always feasible, affordable or sufficient to improve health.^46 47^

Clubs and collectives intentionally engage group or community members in activities that strengthen and leverage interpersonal relationships to address risk factors for ill health. For example, sex worker collectives leveraged bonds between sex workers to create a unified front against violence and discrimination, resulting in increased condom usage and uptake of sexual health services.^48^ In theory, participants in classroom interventions might access such support through their existing relationships with family, friends or neighbours. In many contexts, this is infeasible either due to patriarchal gender norms undermining women’s relationships with household and non-household members^49^ or unequal distributions of social capital disproportionately benefitting the already-wealthy.^50^

It is also theoretically possible that classroom participants support one another to improve health after receiving health education and skills training without prompting. Just as there is little evidence for classroom or club members improving population-level health outcomes through their own initiative, there is also little evidence for this happening. A classroom intervention for members of the Indian trade union SEWA (Self-Employed Women’s Association) to reduce hospitalisation and morbidity through delivery of preventive care information showed no effect.^51^ Even though many members had cooperated with one another over finance, social entitlements and organising for workers’ rights, they did not engage in spontaneous collective action for health when given relevant information.^51^

*Can intervention designers reliably identify a limited set of bottleneck behaviours preventing the health outcome from being achieved?* Classroom and club interventions using a didactic pedagogy must assume such behaviours—along with their enablers and barriers—are known or rapidly identifiable, when they design training programmes and messages to target key behaviours.^21^ Although bottleneck behaviours and their enablers and barriers may be reasonably accurately ascertained through formative research in the context of a pilot or efficacy trial, this is challenging to achieve at scale.^25^ A randomised controlled trial in India showed that an intervention carefully tailored to local context cut neonatal mortality in half.^52^ When this intervention was scaled up to cover a population of 23 million, no evidence for mortality impact was found.^53^

When complexity is involved in solving a health issue, bottleneck behaviours may not exist, not be known or not suffice to improve health outcomes. Experts may not know which behaviours are bottlenecks for a particular context given the time, cost and scientific challenges involved in identifying the magnitude of causal effects of risk factors. Changing one set of risk factors may have minimal impact on health outcomes, when its removal results in the substitution of competing factors.^54^ A nutrition intervention in rural India found impact on key child feeding and care behaviours, but no impact on child morbidity or anthropometry—poor local availability and affordability of nutritious foods and healthcare prevented dietary changes from translating into health improvement.^55^

Collectives and clubs, which take a problem-solving approach to health communication, build on participants’ knowledge of what problems matter and how they can be addressed.^56^ In a trial of participatory learning and action with groups to prevent diabetes in Bangladesh, stigma against diabetes sufferers unexpectedly turned out to be a major barrier to physical activity, as anyone seen exercising in public could be suspected of having diabetes.^57^ Staff and group members secured agreement not to criticise men or women for exercising. Along with improvements in many other care and care-seeking behaviours for diabetes, a 61% reduction in incidence of diabetes was observed after just 2 years.^10^

*Do group or community members have sufficient time and willingness to reflect, innovate and adapt to solve their health problems together with a facilitator?* Collectives and problem-solving clubs need to assume that group and community members are capable of carrying out effective problem diagnosis and solution. Classrooms and didactic clubs only require minimal assumptions about knowledge, creativity, motivation or skill of group members, as these are carefully instructed in the exact behaviours they need to change. Group deliberation is critical to ensuring effective problem diagnosis, as participants build on each other’s capacities to collectively acquire knowledge and skills that they cannot access as individuals. For example, after discussion in groups of 15 to 25 to identify and rank maternal health problems, members of participatory women’s groups in Malawi independently arrived at the same ranking as clinical incidence data from an institutional review of local causes of maternal mortality.^58^

Efforts to encourage group and community members to support one another do not always succeed.^59^ Community members may be insufficiently motivated to take collective action.^60^ An intervention to prevent intimate partner violence in South Africa found many men and women failed to participate in collective action due to time constraints, lack of material incentives and fear of negative community reactions.^61^ When the intervention was evaluated, only impact on group members was found; there was no evidence for a population-level reduction in violence.^13^ Improving the health outcome may also require changes to the health system that are beyond the capacity of a local community to address.^62^ Such outcomes may be better suited to structural interventions supporting coalitions between community members, civil society, health providers and policymakers.^63^

## Conclusion

We presented a simple typology of health interventions with groups to highlight previously unwritten assumptions governing different types of health intervention that likely matter for their eventual impact in specific contexts. We delineated two axes of variation called the style and scope of intervention activities, which we used to characterise three ideal types of intervention with groups, namely the classroom, the club and the collective. We argued population-level health outcomes determined by a complex mix of mutually interacting risk factors, whose relative importance and social causes may be largely unknown to experts, are best addressed through collective interventions. Where interventions seek to impact only on group members’ health, clubs or classrooms may be appropriate depending on the complexity of the health outcome.

We developed our typology to explain clear differences in health impact by intervention type in a recent systematic review of women’s group interventions in India.^7^ Importantly, our review highlighted that different types of women’s groups, such as microfinance-oriented self-help groups or sex workers collectives, can use different intervention ideal types depending on the context. For example, a government-run self-help group programme in Bihar, India, has implemented a classroom intervention to improve dietary diversity,^38^ but also a club intervention to reduce gender-based violence^64^ and a collective approach with participatory learning and action.^65^ Thus, the underlying group does not limit the type of intervention approach that can be implemented to improve health.

Our typology is not without limitations. First, we largely drew on examples of interventions from South Asia and sub-Saharan Africa where most such evaluations have taken place. Second, we caution that our typology only applies to health interventions delivered to groups of laypeople who physically meet on a regular basis. Our arguments are not aimed at one-on-one interventions such as home visits.^66^ Third, our typology is meant to complement, not replace existing typologies of community-based intervention.^67^ Health interventions clearly differ depending on whether they involve new or existing groups, target the general population or a special subpopulation, or involve complementary technological or economic interventions.^59 67^ Our typology is a tool to ensure consideration of pertinent intervention characteristics, not an exhaustive mapping of all sources of variation. Finally, we primarily reviewed evidence on health impact, without which issues of sustainability are moot. A fuller typology in the future could also reflect evidence on sustainability of group-based interventions after external agents have withdrawn.^68^

We invite public health researchers and policymakers to use our framework in their respective contexts. If intervention types are mismatched to health outcomes, funders will either over-invest or fail to achieve the impacts they seek. Seeking to reduce domestic violence with a classroom intervention is unrealistic, given the entrenched environmental barriers, including poor access to justice, stigmatising social norms and lack of agency in the household that perpetuate domestic violence.^69^ Assuming that information transfer to existing financial or livelihoods groups—which may or may not have built internal social capital—can improve complex, multifactorial health problems is not only ambitious, but also ignores the importance of the time and investment required to build successful collective action for health.^70^ We hope that our typology will help researchers and policymakers align their own and their communities’ ambitions with appropriate interventions for health.
